# Stability of Fucoxanthin in Pasteurized Skim and Whole Goat Milk

**DOI:** 10.3390/foods10071647

**Published:** 2021-07-16

**Authors:** Maryuri T. Nuñez de González, Rahmat Attaie, Adela Mora-Gutierrez, Selamawit Woldesenbet, Yoonsung Jung

**Affiliations:** Cooperative Agricultural Research Center, Prairie View A&M University, Prairie View, TX 77446, USA; mtnunez@pvamu.edu (M.T.N.d.G.); admora@pvamu.edu (A.M.-G.); sewoldesenbet@pvamu.edu (S.W.); yojung@pvamu.edu (Y.J.)

**Keywords:** goat milk, fucoxanthin, pasteurization, storage time

## Abstract

Obesity has become a worldwide problem giving rise to several health issues. Fucoxanthin, a marine carotenoid with anti-obesity activity, has potential application as a biofunctional ingredient in human food. The objective of this study was to evaluate the thermal stability of fucoxanthin at pasteurization temperature and, subsequently, its storage stability in goat whole milk (WM) and skim milk (SM) at refrigeration temperature for four weeks. Additionally, the effect of supplementation of fucoxanthin on the composition of milk, pH, acidity, color, and lipid oxidation of WM and SM was evaluated during the four week storage period. Fresh goat WM and SM were supplemented with fucoxanthin at a concentration of 10.67 µg/mL (2.56 mg/240 mL of milk, one serving), pasteurized at 64 °C for 30 min and stored at 4 °C for four weeks. The quantification of fucoxanthin in WM and SM was performed every week using a HPLC method. Moreover, the effect of supplementation of fucoxanthin on the composition of WM and SM was evaluated by a LactiCheck milk analyzer, and the color was evaluated by reflectance using a HunterLab colorimeter. Lipid oxidation, as the 2-thiobarbituric acid-reactive substances (TBARS) at A_532_, was determined using a Spectramax Plus spectrophotometer during storage. Data were analyzed by a split-plot design using PROC MIXED of SAS. The recovery yields of fucoxanthin from the pasteurized WM and SM were 96.17 ± 1.5 % and 96.89 ± 1.5 %, respectively. Both milks exhibited high recovery yields of fucoxanthin. Fucoxanthin was stable in goat WM and SM during storage at 4 °C for four weeks. The addition of fucoxanthin, at the concentration reported to have an anti-obesity effect in humans, to pasteurized WM and SM did not affect the composition or the physicochemical properties of milks but influenced the color, especially increasing the yellowness in the samples. These results revealed that goat milk can be used as a suitable matrix for the supplementation of fucoxanthin as a biofunctional ingredient in human foods.

## 1. Introduction

Consumers have an increased interest in nutrition as foods have been linked to health and wellness, primarily in the control of obesity. Obesity is associated with some of the most costly and serious health problems, such as type-2 diabetes, cardiovascular diseases, and metabolic syndrome [1]. Based on the National Health and Nutrition Survey (NHANES), the prevalence of obesity in U.S. youth and adults was 18.5 and 39.8%, respectively, in 2015–2016 [2]. Since obesity continues to be an important health problem, developing effective preventive measures to reduce obesity and ease the medical and economic burden of obesity-related diseases such as cancer, heart disease, and diabetes, which amounts to billions of dollars annually, is essential. Nutrition can play a major role in preventing these lifestyle-related diseases and it is highly desirable to find safe and effective biofunctional ingredients in food to mitigate these health-related issues [3].

The importance of marine algae as sources of functional ingredients has been well recognized due to their beneficial health effects. Among functional ingredients identified from marine algae, fucoxanthin has received particular interest. This carotenoid, extracted from marine brown seaweeds, microalgae, and diatoms, has been reported to exhibit anti-obesity, anti-diabetic, and anti-inflammatory bioactivities [4,5]. 

The anti-obesity effect of fucoxanthin has been reported in several studies [6,7,8,9,10,11]. This anti-obesity effect has been closely associated with the unique structure of fucoxanthin, which has an allene bond and an additional hydroxyl substituent on the side group of the fucoxanthin metabolites, fucoxanthinol and amarouciaxanthin A [12]. Fucoxanthin, a marine carotenoid with non-provitamin A xanthophyll, induces the mitochondrial uncoupling protein 1 expression in white adipose tissue (WAT), causing the oxidation of fatty acids and heat production in WAT [5,13]. In a study to evaluate the anti-obesity effect of fucoxanthin, Maeda et al. [14] found that the uncoupling protein 1 was clearly expressed in the WAT of obese mice that were fed fucoxanthin at 0.1% concentration. In a dietary study using mice, it was reported that a high-fat with fucoxanthin-rich wakame lipid diet significantly suppressed body weight and WAT weight gain induced by the high fat diet [15]. The combination of fucoxanthin and conjugated linoleic acid has been shown to exert an anti-obesity effect in high fat diet-induced obese rats by regulating mRNA expression of enzymes associated with lipid metabolism in the WAT [16]. 

In a clinical study, Abidov et al. [17] found that a dose of 2.4 mg/day of fucoxanthin resulted in increased energy expenditure in the body and significant weight loss after 16 weeks of diet in obese premenopausal women that were non-diabetic with non-alcoholic fatty liver disease and normal liver fat. Likewise, Hitoe and Shimoda [18] studied the effect of fucoxanthin capsules (3 mg/day) on male and female Japanese adults with a body mass index of more than 25 kg/m^2^ in a clinical trial for four weeks. Their results indicated a reduction in body weight, BMI, and abdominal fats. Brown algae or brown seaweeds that contain fucoxanthin are generally recognized as safe (GRAS) in foods as flavor enhancers and flavor adjuvants, according to the Code of Federal Regulations [19]. 

Carotenoids are highly sensitive to environmental stressors such as oxygen, light, heat, and pro-oxidant metal ions [20]. Milk, due to its natural emulsifying capacity, is an effective vehicle for liposoluble micronutrients such as the carotenoids (i.e., fucoxanthin, astaxanthin, β-carotene, lycopene, and lutein). Mok et al. [21] demonstrated that cow whole milk or skim milk can be used as a basic food matrix for fucoxanthin application and that protein content in milk is a major factor for fucoxanthin stability. Additionally, fucoxanthin bioavailability in vivo and in vitro from fucoxanthin-fortified cow whole milk and skim milk was investigated [22]; the results of this study showed that cow skim milk was a good food matrix for fucoxanthin application in terms of its stability and bioavailability. Since cow milk constituents, i.e., proteins and fat, bind strongly to the carotenoids as was demonstrated by Mok et al. [21,22], we expected that goat milk caseins would be more effective in preventing the chemical degradation of fucoxanthin than cow milk caseins, due to its higher content of β-casein. Beta-casein is highly hydrophobic in nature and, thus, could form a thick protective layer preventing oxidation of fucoxanthin and enhance the chemical stability of this carotenoid [23].

Therefore, we expected that fucoxanthin supplemented into goat milk and milk products would be bioactive and stable at pasteurization temperature and during their storage under refrigerated conditions. Moreover, it was expected that supplementation of fucoxanthin into goat milk products would not negatively affect their overall physicochemical attributes during production and storage. To our knowledge, no research has been conducted on the stability of fucoxanthin in goat milk products or how it can affect the physicochemical characteristics of these products. Thus, the objective of this study was to evaluate the stability of fucoxanthin after pasteurization and during storage in goat WM and SM at 4 °C for four weeks. Furthermore, the physicochemical characteristics of fucoxanthin supplemented goat WM and SM after pasteurization and during the storage period were also studied.

## 2. Materials and Methods

### 2.1. Materials

Food grade fucoxanthin with 20% purity was purchased from Shandong Jiejing Group Corporation (Rizhao, China). All chemicals and reagents used were of analytical grade or HPLC grade. Fucoxanthin standard (99.5% purity), phenolphthalein, trichloroacetic acid, 2-thiobarbituric acid (TBA), sodium hydroxide (NaOH), tert-butyl methyl ether (TBME), and methanol were purchased from Sigma-Aldrich (St. Louis, MO, USA). Ethanol (200 proof) and petroleum ether were purchased from Fisher Scientific (Billerica, MA, USA). Deionized water, prepared by passing distilled water over a mixed bed of cation-anion exchanger, was used throughout this study. 

### 2.2. Goat Milk Collection and Preparation 

**Figure 1:**
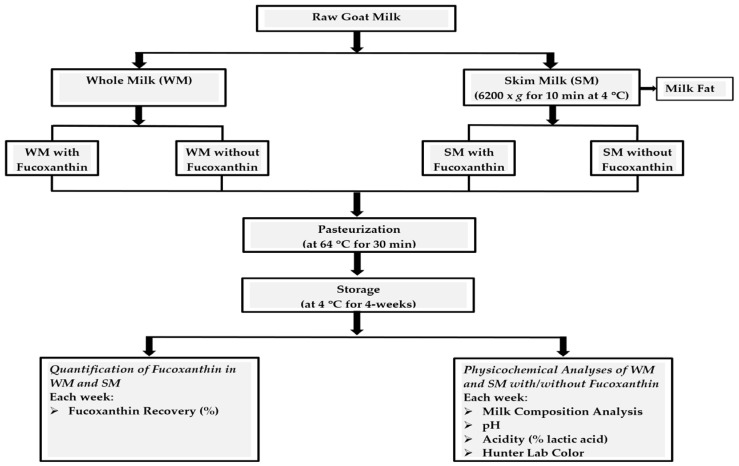
Flow chart of goat milk processing and physicochemical analyses.

Three replicates of approximately six liters of milk from Alpine goats were collected from the bulk tank of the milking parlor at the International Goat Research Center at Prairie View A&M University. Fresh goat milk was brought to the laboratory and divided into two batches of WM and SM. The goat skim milk was prepared by centrifugation (Avanti J-E centrifuge, Beckman Coulter Inc., Indianapolis, IN, USA) using 100 mL centrifuge tubes at 6200× *g* at 4 °C for 10 min. The fucoxanthin with 20% purity was dissolved into HPLC grade ethanol and mixed with both goat raw whole and skim milk to a final concentration of 10.67 μg/mL (2.56 mg/240 mL of milk, which is one serving). Samples of WM and SM without fucoxanthin were used as the controls. Both raw WM and SM were pasteurized by LTLT (low temperature—long time) at 64 °C for 30 min (Safgard Pres-Vac Home Pasteurizer Model P-3000; the Schlueter Co., Janesville, WI, USA). The samples of whole or skim milk with and without fucoxanthin were stored under refrigeration temperature (4 °C) for storage stability studies. All samples that were used for the analyses of milk composition, quantification of fucoxanthin, determination of pH and titratable acidity, evaluation of color, or the measurement of lipid oxidation were pasteurized by LTLT. A flow chart of goat milk processing and physicochemical analyses is given in Figure 1.

### 2.3. Quantification of Fucoxanthin from Goat Milk

Extraction and quantification of fucoxanthin from the raw and pasteurized whole and skim milk were carried out according to the method of Mok et al. [21] with slight modifications. Briefly, exactly 2 mL samples of either WM or SM that contained fucoxanthin were transferred into 10 mL (Pyrex) test tubes and 2 mL of ethanol was also added for deproteination. One milliliter of petroleum ether and then 1 mL of tert-butyl methyl ether (TBME) were added to the extraction tube and the content was mixed (vortex) for 30 s. The mixture was centrifuged (Avanti J-E centrifuge, Beckman Coulter Inc., Indianapolis, IN, USA) at 3500 rpm (1838 RCF× *g*) for 5 min to extract fucoxanthin from the food matrix. The supernatant was collected into a 15 mL test tube. The addition of petroleum ether and TBME was repeated three times and the supernatant was collected each time after the centrifugation step. All the collected supernatants from each sample were placed into a nitrogen evaporator (N-Evap Model 111, Organomation Associates, Inc., Berlin, MA, USA) for drying under nitrogen gas. The water bath temperature of the nitrogen evaporator was set at 30 °C and the flow rate of nitrogen gas was set at 2 L/min. The dried samples were dissolved into exactly 1 mL of 90% aqueous ethanol and then transferred by 1 mL-plastic syringe (Norm-Ject^®^, Ace Glass Inc., Vineland, NJ, USA) using an 18-gauge needle (BD Precision Glide, Franklin Lakes, NJ, USA). The needle was removed and an Acrodisc 25 mm syringe filter with a 0.45 μm membrane (HT Tuffryn Membrane; Life Sciences, Lake Mary, FL, USA) was placed at the tip of the syringe. The extract was filtered into HPLC vials before analysis. The WM samples with fucoxanthin went through one more extraction step. One milliliter of 90% ethanol was added to the dried whole milk extract and then 1 mL of n-hexane for removal of milk fat was added to the extract. The 1 mL of n-hexane with milk fat was removed from the mixture and the sample was completely dried. The dried sample was dissolved into exactly 1 mL of 90% ethanol and was used for HPLC analysis after filtration with a 0.45 μm membrane as previously stated. 

Quantification of fucoxanthin was performed using HPLC system (1260 Infinity, Agilent Technologies, MA, USA) and a YMC C-30 carotenoid column (250 × 4.6 mm i.d., 3 µm particle size, Waters, MA, USA). The mobile phase consisted of a methanol and water solvent system with a flow rate of 0.7 mL/min and column temperature of 35 °C. The following solvent gradient program was used: methanol/water ratio was increased from 90:10 to 100:0 over a 20 min period, and then 100% methanol was run for the last 5 min. The chromatogram obtained at 450 nm was used for quantitative analysis of fucoxanthin. 

The analytical grade fucoxanthin stock solution (4 mg/mL with 99.5% purity, Sigma-Aldrich, St. Louis, MO, USA) was used to obtain concentrations of 4, 8, 12, and 16 μg/mL. The diluted concentrations were used to construct the standard curve. The diluted concentrations were injected into the HPLC system under the same running conditions as mentioned above to construct the standard curve. The standard curve (*Y* = a*x* + b) with the constant values of *Y* = 24.529 × *x* − 8.4328 (*R*^2^ = 0.9989) was used to calculate the quantities of fucoxanthin in each sample. The samples were analyzed in triplicate and the fucoxanthin recovery percent was reported.

### 2.4. Composition of Pasteurized Goat Milk

The percentages of fat, solids-not-fat (SNF), total solids (TS), and protein in pasteurized WM and SM samples with or without fucoxanthin (the controls) were determined using the LactiCheck ultrasonic milk analyzer (Model LC-02, Page and Pedersen International Ltd., Hopkinton, MA, USA) according to the recommended procedure. The LactiCheck Ultrasonic milk analyzer was calibrated with pre-assayed UHT milk standards containing 2% and 3.5% milk fat at the onset of measurements every time the unit was used. The lactiCheck Ultrasonic milk analyzer measurement ranges for the percentage of fat, SNF, and protein were 0.3–9.0%, 6–12%, and 2–5%, respectively. Sample analyses of WM and SM that were supplemented with fucoxanthin or without fucoxanthin and stored at 4 °C were carried out every week for four weeks. Each pasteurized milk sample containing fucoxanthin or without fucoxanthin was analyzed in triplicate on weeks 1, 2, 3, and 4 of storage, and the percentages of milk components were reported.

### 2.5. Determination of pH and Titratable Acidity

The pH of WM and SM samples with fucoxanthin and their controls without fucoxanthin was determined throughout the four week storage period using an Accumet benchtop pH meter (Fisherbrand™ Accumet™ AE150, Fisher Scientific, Pittsburgh, PA, USA) at 22 °C. The reference buffers (4.00 and 7.00; Orion buffer solutions; Thermo Fisher Scientific, Pittsburgh, PA, USA) that were used for the calibration of pH meter and the analyzed milk samples were at room temperature (22 °C). The titratable acidity in the milk samples was determined according to the AOAC method No 947.05 [24]. Briefly, to each 20 mL sample of goat milk, a 40 mL of boiled and cooled distilled water was added. Phenolphthalein (1% *w*/*v* in 95% ethanol) was used as an indicator. The mixture was titrated with standardized 0.1 N NaOH until the first color change, which signals the endpoint (at pH 8.1–8.3), persisted for 30 s. One more drop of 0.1 N NaOH was added and the final volume of NaOH used for titration was recorded. The results were expressed in percentage of lactic acid. The pH and titratable acidity in the samples were determined in triplicate.

### 2.6. Evaluation of Color in Milk

The effect of supplementation of fucoxanthin on the color of WM and SM, during the four weeks of storage at 4 °C, were determined by reflectance using a HunterLab colorimeter (ColorFlex^®^ Spectrophotometer, Hunter Associates Laboratory, Inc., Reston, VA, USA). The colorimeter was calibrated using a white tile with D65/10° Illuminant/observer. The color of each milk sample supplemented with fucoxanthin or without fucoxanthin was determined in triplicate at room temperature (~22 °C) for weeks 1, 2, 3, and 4 of storage. The L* (lightness), a* (red-green) and b* (blue-yellow) values were reported.

### 2.7. Measurement of Lipid Oxidation in Milk

Lipid oxidation of whole and skim milk samples with or without fucoxanthin was measured each week following the procedure of King [25] during the four weeks of storage at 4 °C. Milk samples (17.6 mL) were pre-warmed to 30 °C and precipitated by adding 1 mL of trichloroacetic acid solution (1 g/mL) and 2 mL of 95% ethanol. The samples were shaken vigorously for 10 s and then incubated for 5 min at 30 °C. After incubation, the precipitate was filtered using a Whatman filter paper grade 42 (Whatman^®^, Sigma-Aldrich, St. Louis, MO, USA). One milliliter of 1.4% 2-thiobarbituric acid (TBA) was added to 4.0 mL of the clear filtrate and the mixture was shaken for 10 s. Finally, the mixture was incubated for 1 h at 60 °C, cooled for 10 min, and the absorbance was measured at 532 nm in a Spectramax Max Plus spectrophotometer (Molecular Devices, Sunnyvale, CA, USA). A fresh solution of TBA was prepared every week for the analysis of 2-thiobarbituric acid-reactive substances (TBARS). All determinations were performed in triplicate and the results were expressed as TBARS.

### 2.8. Statistical Analysis

Experimental data were analyzed as a split-plot design using the PROC MIXED model procedure of SAS (version 9.4, SAS Institute Inc., Cary, NC, USA). For fucoxanthin recovery data during the storage time, the whole plot was the effect of product type (goat WM and SM with fucoxanthin) and the split-plot was storage week. For the physicochemical parameters in pasteurized WM or SM, the whole plot was ingredient effect and the split-plot was storage week. The treatment (milk with FX and control-without FX) and storage weeks (1, 2, 3, and 4) were the main effects of the experiment. 

A factorial design with two factors (milk type and replicate) was used for fucoxanthin data to evaluate the recovery technique of a known amount of fucoxanthin into milk samples. In addition, a factorial design with two factors (milk treatment and milk type) was used to evaluate the stability of fucoxanthin after pasteurization. Analysis of variance was used to determine statistical differences among the means of the main effects and their interactions and considered significant at *p* < 0.05. A total of three replicates were performed and three samples from each treatment of a replicate were analyzed. The least-square means and their standard errors (SE) were used to identify significant differences between treatments. 

## 3. Results and Discussion

The extraction and recovery technique that was used for the analysis of fucoxanthin in goat WM and SM was accurate. The recovery procedure of fucoxanthin from WM and SM with a known amount of fucoxanthin (10.67 µg/mL) in each sample was assayed using four replicates and three samples from each replicate. The overall results indicated that the percent recovery of fucoxanthin was high, 96.17 and 96.89 in WM and SM, respectively (Table 1).

The results of heat stability (Table 2) indicated that the mean values for percent recovery of pasteurized milks and the controls did not differ (*p* > 0.05). Raw WM and SM were used for comparison as the controls. The percent recovery of fucoxanthin was not different (*p* > 0.05) when compared between goat WM and SM. The stability of fucoxanthin after the heat of pasteurization was excellent and was nearly the same as the maximum percent of recovery (Table 1). These results indicated that fucoxanthin was not affected by the heat of LTLT pasteurization in either goat WM or SM.

Similarly, fucoxanthin was stable during the storage period of goat WM and SM at refrigeration temperature (4 °C). The results indicated that the overall mean stability of fucoxanthin in both milks after four weeks of storage (Table 3) was close to the maximum percent of recovery (Table 1). The mean values of percent recovery of fucoxanthin after each week of storage were not different (*p* > 0.05) for either WM or SM treatments at refrigeration temperature. Additionally, the mean values of percent recovery during the storage period were not different (*p* > 0.05) between the WM and SM, indicating that fucoxanthin was stable at refrigeration temperature for four weeks.

In this study, the fucoxanthin recovery values from goat WM and SM were slightly higher than those values reported by Mok et al. [21] for pasteurized cow milk (at 65 °C for 30 min). Those researchers reported fucoxanthin recovery values of 95.37 ± 1.06% for cow WM and 93.25 ± 0.76% for cow SM. Since fucoxanthin is a lipophilic carotenoid and quickly partitions into the lipid phase of goat milk, a thorough mixture of milk content is required for drawing a representative sample from each container, and this is particularly important in the case of whole milk. In this study, the milk containers were heated to 38 °C and thoroughly mixed prior to taking samples. The accuracy of data dictates that representative samples should be quickly drawn from each milk container before partitioning of fucoxanthin into the lipid phase of milk. 

### 3.1. Effect of Fucoxanthin on Physicochemical Properties of Pasteurized Goat Whole Milk 

The results of milk composition, pH, and acidity of pasteurized goat WM without fucoxanthin (control) and with fucoxanthin are presented in Table 4. The percentages of fat, protein, SNF, and TS did not differ (*p* > 0.05) between the treatment and the control. Likewise, the values of pH and acidity were not affected (*p* > 0.05) by the addition of fucoxanthin in goat WM when compared to the control. Additionally, the values of these parameters in the treatment and the control were not affected (*p* > 0.05) by storage time.

The supplementation of goat WM with fucoxanthin had a significant effect (*p* < 0.05) on a* (red-green) and b* (blue-yellow) color space values but not on the L* (lightness) values (Table 5). The redness (positive a*) and yellowness (positive b*) space values were higher (*p* < 0.05) for the fucoxanthin supplemented milk samples as compared to the control. The marked increase in yellowness is due to the natural pronounced orange color of fucoxanthin pigments. Similarly, O’Sullivan et al. [26] found significantly higher a* and b* values in milk samples by the addition of seaweed extracts. These authors indicated that the higher b* values or yellowness may be due to the presence of yellow pigments such as fucoxanthin, rutin, or morin in the seaweed extract. As shown in Table 5, no differences (*p* > 0.05) were noted in the lipid oxidation, measured as TBARS, between milk samples containing fucoxanthin and the control. Moreover, no significant differences (*p* > 0.05) were observed in these physicochemical parameters in fucoxanthin supplemented and pasteurized goat WM due to the effect of storage time (Table 5) or the treatment and storage time interactions (data not shown).

### 3.2. Effect of Fucoxanthin on Physicochemical Properties of Pasteurized Goat Skim Milk 

In comparison with the control, the inclusion of fucoxanthin into goat SM did not have any impact on the composition of milk, pH, and acidity (Table 6), except that the fat content was higher (*p* < 0.05) in pasteurized SM with fucoxanthin. This may be due to the fact that the fucoxanthin used in this experiment had only 20% purity. A slight amount of fat among the 80% impurity of fucoxanthin can change the percentage of fat content in milk, particularly in SM where the fat content is very low. The legal limit of fat in skim milk is 0.5% or less in the United States and our SM had less than 0.5%, which meets the limit. Since the fat values are generally small, a slight change in the fat content can change the values statistically. However, the real life-effect of such a small change in the fat content of milk is not pronounced in the human diet. The values of other parameters in fucoxanthin supplemented SM did not change (*p* > 0.05) due to storage time (Table 6).

On the other hand, supplementation of fucoxanthin changed (*p* < 0.05) the color attributes of the goat SM samples by decreasing the lightness (slightly darkening the samples), and increasing the redness and yellowness (Table 7). The yellowness was the color attribute that was most impacted by the addition of fucoxanthin in SM due to the natural orange color of fucoxanthin pigments. There was no significant change (*p* > 0.05) in the values of the color parameters of pasteurized SM due to storage time (Table 7) or the treatment and storage time interactions (data not shown).

Overall, the supplementation of SM with fucoxanthin did not affect the percent composition of milk; however, a change in the product color was observed due to the natural color of fucoxanthin pigments. In addition to being a very important bioactive compound with anti-obesity characteristics, the coloring property of fucoxanthin in some instances can be useful as a natural colorant, in particular to those goat dairy products that are preferred as yellow-orange such as Cheddar or American cheese, butter, yogurt, ice cream, and fruit-flavored milks (e.g., passion fruit, apricot, peach, and mango). 

Future studies will focus on the sensory quality attributes of fucoxanthin supplemented goat WM and SM as well as the evaluation and stability of fucoxanthin in different types of goat dairy products such as cheese, yogurt, and ice cream. Moreover, the in vivo and in vitro bioavailability, and the anti-obesity effect, of fucoxanthin supplemented goat dairy products will be investigated in human subjects.

## 4. Conclusions

The percentage recovery of fucoxanthin from the supplemented and pasteurized goat WM and SM was high, indicating that fucoxanthin was stable at the heat of pasteurization (64 °C for 30 min) and was not degraded. Additionally, fucoxanthin was stable in these products during the four weeks of refrigerated storage at 4 °C. Fucoxanthin, used as a biofunctional ingredient in goat WM and SM, did not affect the composition, pH, acidity, or lipid oxidation of these products under the experimental conditions of this study. However, the supplementation of fucoxanthin in both pasteurized goat milks increased the redness and yellowness. The intensity of yellow color was higher in goat SM than WM. In general, the physicochemical characteristics of WM and SM were not affected by the addition of fucoxanthin. Based on these results, both goat WM and SM can be used as effective carriers of this carotenoid as a biofunctional ingredient with an anti-obesity effect, and for the development of several functional dairy products for human consumption. 

## Figures and Tables

**Table 1:** The percent recovery ^1^ of known amount of fucoxanthin spiked into pasteurized goat whole and skim milk.

Type of Milk	Replicate				Overall Recovery
	1	2	3	4	
Whole milk	101.02 ± 2.87	97.93 ± 2.87	94.29 ± 2.87	91.42 ± 2.87	9617 ± 1.5
Skim milk	96.19 ± 2.87	95.22 ± 2.87	98.56 ± 2.87	97.60 ± 2.87	96.89 ± 1.5

^1^ Least square mean values with their standard errors. The initial concentration of fucoxanthin spiked into milks (10.67 μg/mL) was considered as 100%.

**Table 2:** Stability of fucoxanthin based on percent recovery ^1^ after heat of pasteurization in goat whole and skim milk.

Milk Treatment	Type of Milk	
	Skim Milk	Whole Milk
Raw milk	95.75 ± 1.25	94.50 ± 0.82
Pasteurized LTLT ^2^	96.16 ± 1.18	93.65 ± 1.28

^1^ Least square mean values with their standard errors. The initial concentration of fucoxanthin into milks (10.67 μg/mL) was considered as 100%. ^2^ LTLT; low temperature-long time (64 °C for 30 min).

**Table 3:** Stability of fucoxanthin based on percent recovery ^1^ in goat whole and skim milk during storage at refrigeration temperature (4 °C).

Type of Milk	Storage Time (Week)				Overall Mean
	1	2	3	4	
Whole milk	97.59 ± 1.73	98.08 ± 1.73	94.17 ± 1.73	93.74 ± 1.73	95.90 ± 1.20
Skim milk	98.85 ± 1.73	93.87 ± 1.73	97.41 ± 1.73	94.20 ± 1.73	96.08 ± 1.20

^1^ Least square mean values with their standard errors. The initial concentration of fucoxanthin into milks (10.67 μg/mL) was considered as 100%.

**Table 4:** Effect of fucoxanthin supplementation on goat whole milk composition, pH, and acidity ^1^ during storage (4 °C).

Parameter	Treatment ^2^			Storage Time (Week)				
	Control (*n* = 24)	FX (*n* = 24)	SE	1 (*n* = 12)	2 (*n* = 12)	3 (*n* = 12)	4 (*n* = 12)	SE
Milk composition (%)								
Fat	3.43	3.23	0.19	3.12	3.53	3.61	3.07	0.19
Protein	3.43	3.49	0.17	3.34	3.51	3.48	3.51	0.17
Solids-no-fat	9.11	9.34	0.43	8.86	9.32	9.23	9.49	0.43
Total solids	12.54	12.57	0.60	11.97	12.85	12.83	12.55	0.61
pH	6.60	6.65	0.08	6.61	6.59	6.64	6.67	0.08
Acidity (% lactic acid)	0.20	0.19	0.05	0.20	0.19	0.21	0.19	0.05

^1^ Least square mean values with their standard errors (SE). ^2^ Control, pasteurized milk without fucoxanthin; FX, pasteurized milk with fucoxanthin at 10.67 μg/mL concentration.

**Table 5:** Effect of fucoxanthin supplementation on color and lipid oxidation ^1^ of goat whole milk during storage (4 °C).

Parameter	Treatment ^2^			Storage Time (Week)				
	Control (*n* = 24)	FX (*n* = 24)	SE	1 (*n* = 12)	2 (*n* = 12)	3 (*n* = 12)	4 (*n* = 12)	SE
HunterLab color								
L*	89.11	82.61	1.05	85.66	86.02	86.06	85.70	0.89
a*	−2.69 ^b^	4.87 ^a^	0.87	1.30	1.18	1.28	0.61	0.62
b*	7.60 ^b^	36.44 ^a^	4.40	22.78	22.12	22.70	20.50	3.12
TBARS (at A_532_)	0.03	0.02	0.00	0.02	0.02	0.03	0.03	0.00

^1^ Least square mean values with their standard errors (SE). ^2^ Control, pasteurized milk without fucoxanthin; FX, pasteurized milk with fucoxanthin at 10.67 μg/mL concentration. ^a,b^ Means in the same row within each effect (treatment or storage time) with different superscripts differ (*p* ˂ 0.05).

**Table 6:** Effect of fucoxanthin supplementation on goat skim milk composition, pH, and acidity ^1^ during storage (4 °C).

Parameter	Treatment ^2^			Storage Time (Week)				
	Control (*n* = 24)	FX (*n* = 24)	SE	1 (*n* = 12)	2 (*n* = 12)	3 (*n* = 12)	4 (*n* = 12)	SE
Milk composition (%)								
Fat	0.44 ^b^	0.56 ^a^	0.02	0.49	0.50	0.50	0.48	0.02
Protein	3.50	3.69	0.40	3.60	3.60	3.59	3.60	0.40
Solids-no-fat	9.32	9.87	1.06	9.60	9.62	9.58	9.59	1.05
Total solids	9.77	10.43	1.04	10.10	10.14	10.08	10.07	1.04
pH	6.78	6.79	0.01	6.79	6.75	6.79	6.82	0.01
Acidity (% lactic acid)	0.15	0.15	0.02	0.15	0.15	0.18	0.12	0.02

^1^ Least square mean values with their standard errors (SE). ^2^ Control, pasteurized milk without fucoxanthin; FX, pasteurized milk with fucoxanthin at 10.67 μg/mL concentration. ^a,b^ Means in the same row with different superscripts differ (*p* ˂ 0.05).

**Table 7:** Effect of fucoxanthin supplementation on color and lipid oxidation ^1^ of goat skim milk during storage (4 °C).

Parameter	Treatment ^2^			Storage Time (Week)				
	Control (*n* = 24)	FX (*n* = 24)	SE	1 (*n* = 12)	2 (*n* = 12)	3 (*n* = 12)	4 (*n* = 12)	SE
HunterLab color								
L*	84.10 ^a^	75.51 ^b^	3.11	79.80	80.15	79.70	79.56	3.11
a*	−4.09 ^b^	8.08 ^a^	0.43	1.90	2.18	2.02	1.87	0.43
b*	5.69 ^b^	42.56 ^a^	4.25	24.16	24.54	24.26	23.54	3.01
TBARS (at A_532_)	0.03	0.02	0.00	0.02	0.02	0.03	0.03	0.00

^1^ Least square mean values with their standard errors (SE). ^2^ Control, pasteurized milk without fucoxanthin; FX, pasteurized milk with fucoxanthin at 10.67 μg/mL concentration. ^a,b^ Means in the same row within each effect (treatment or storage time) with different superscripts differ (*p* ˂ 0.05).

## Data Availability

The data presented in this study are available on request from the corresponding author (Rahmat Attaie).
